# Reactivation of Old Scars in an Elderly Man Revealing Löfgren's Syndrome

**DOI:** 10.1155/2013/736143

**Published:** 2013-03-04

**Authors:** Vishnu Vardhan Reddy Munagala, Vaishali Tomar, Amita Aggarwal

**Affiliations:** ^1^Consultant Clinical Immunologist & Rheumatologist, Vizag Rheumatology & Immunology Centre, Visakhapatnam 530002, India; ^2^Consultant Radiologist, Vijaya Medical Centre, Visakhapatnam 530002, India; ^3^Department of Clinical Immunology, Sanjay Gandhi Postgraduate Institute of Medical Sciences, Lucknow 226 014, India

## Abstract

Here, we report the case of a 55-year-old man with reactivation of old cutaneous scars associated with a febrile illness, episcleritis, polyarthralgias, erythema nodosum and hilar adenopathy. High-resolution computed tomography (HRCT) revealed right paratracheal, bilateral hilar, and subcarinal lymphadenopathy without any nodular densities in both lung fields. A scar biopsy revealed multiple noncaseating granulomas and confirmed the diagnosis of sarcoidosis. A short course of oral steroids led to regression of systemic symptoms, and the scars returned to baseline size. This patient represented a rare case of simultaneous Löfgren's syndrome and scar sarcoidosis.

## 1. Introduction 

Sarcoidosis is a multisystem granulomatous disease of unknown cause. The diagnosis usually requires a compatible clinical picture, histologic demonstration of noncaseating granulomas, and exclusion of other diseases capable of producing similar histology or clinical features [1]. Systemic * *symptoms* * such* * as * *fatigue,* * night* * sweats,* * and * *weight* * loss are * *common;* * the* * organ * *system * *that * *is* * most * *affected* * varies* * with* * the * *given * *patient [2]. The differential diagnosis of skin lesions in a patient with acute febrile illness and joint pains and hilar lymphadenopathy is extensive. We report a case of Löfgren's syndrome associated with scar sarcoidosis.

## 2. Case Presentation

**Figure 1:**
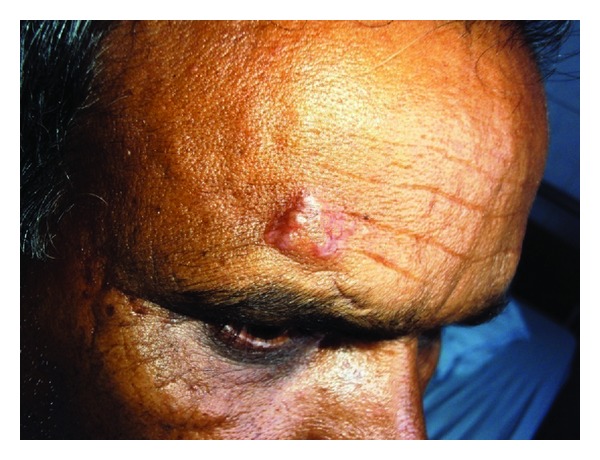
* * Scar enlarged on the forehead.

A 55-year-old man presented to our hospital with 6-week history of low-grade intermittent fever, malaise, fatigue, joint pains involving both knees and ankles along with painful nodules on left lower limb, and redness of both eyes of 1-week duration. He had loss of appetite and a weight loss of 3 kg over this period. He complained of increasing size of old scars present on his forehead (Figure 1). These scars occurred following a road traffic accident 10 years ago. He denies any pruritus or pain in scars. There was no history of breathlessness, chest pain, or night sweats.

Physical examination revealed the following: pulse rate—84/minute, blood pressure—120/80 mm Hg, and respiratory rate—18/minute. The lesions on the forehead were subcutaneous, firm, nontender, and minimally mobile, and he also tender subcutaneous nodules on left shin suggestive of erythema nodosum. Eye examination revealed bilateral episcleritis. He had tenderness in both knees and ankles without any swelling. There were no lymph nodes or hepatosplenomegaly. Chest and cardiac examination was unremarkable.

**Figure 2:**
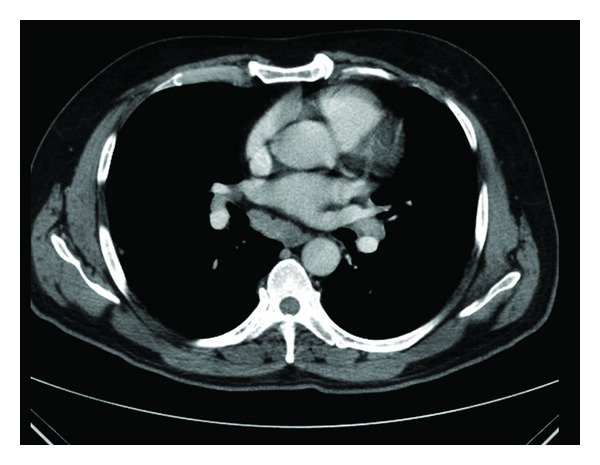
Bilateral hilar and subcarinal lymphadenopathy.

**Figure 3:**
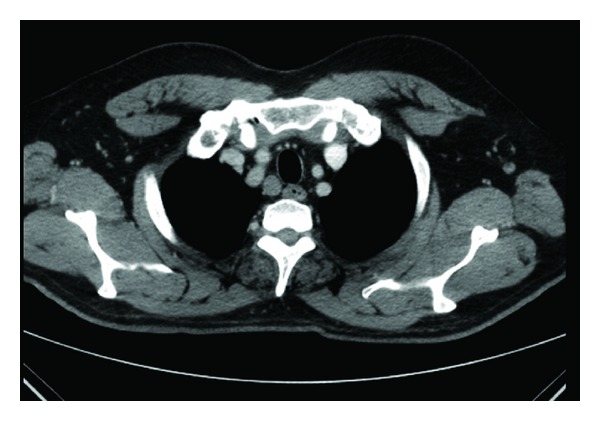
Right Paratracheal lymphadenopathy.

Laboratory investigations showed the following: hemoglobin 14.0 Gm%, TLC of 10800 with 70% polymorphs, 29% lymphocytes, platelet count of 3.06 × 10^9^/L, serum creatinine 1.2 mg/dL, bilirubin 0.3 mg/dL, ALT 51 IU, AST 36 IU, and alkaline phosphatase 161 units. Erythrocyte sedimentation rate was 70 mm/h, and serum calcium was 10.8 mg/dL (normal: 8.5–10.8 mg/dL). A chest radiograph showed doubtful hilar lymph node. High-resolution computed tomography (HRCT) (revealed right paratracheal, both hilar, and subcarinal lymphadenopathy without any nodular densities in both lung fields (Figures 2 and 3). C-reactive protein levels was 96 mg/L. Rheumatoid factor was negative. Antinuclear antibody was 1+ speckled positive at 1 : 100 dilution by indirect immunofluorescence using hep-2 cells, and serum angiotensin-converting enzyme (ACE) level was 17 IU/L (normal: 65–114 IU/L). Tuberculin skin test was negative. ASO titer was <200. Ophthalmoscopic examination, including a slit-lamp study, revealed episcleritis bilaterally.

**Figure 4:**
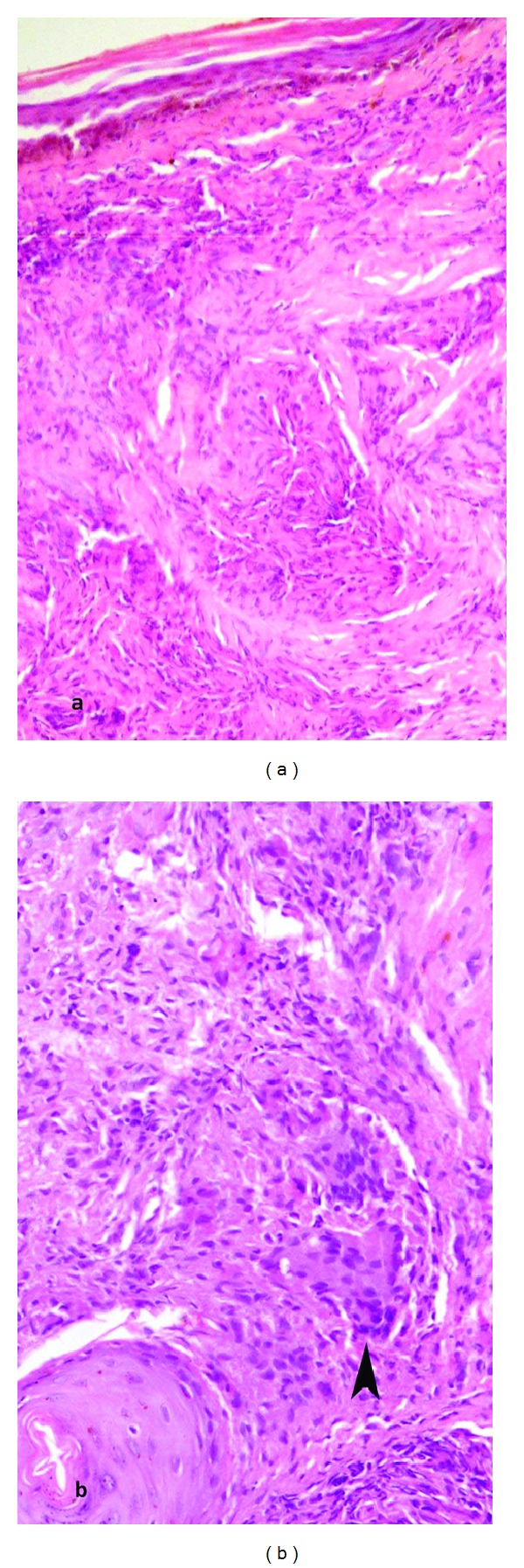
Skin biopsy shows numerous well-circumscribed, noncaseating epithelioid granulomas in the dermis (a) (hematoxylin and eosin, ×200). Few multinucleated giant cells are also seen in the granulomas (b) (arrow head, hematoxylin and eosin, ×400).

A possibility of acute sarcoidosis (Löfgren syndrome), lymphoma, and tuberculosis was considered. The scar on the forehead was biopsied which revealed a noncaseating epitheloid granuloma which was negative for acid-fast bacilli staining, and thus a diagnosis of sarcoidosis was confirmed (Figure 4).

The patient responded to a short course of 10 mg prednisolone daily. His systemic features disappeared, and the enlarged scar resolved to its original size.

## 3. Discussion 

Löfgren's syndrome, an acute form of sarcoidosis characterized by erythema nodosum, bilateral hilar adenopathy, and polyarthralgia or polyarthritis,* * occurs in 9% to * *34%   * *of * *patients [3]. Its presentation is different in men* * and women.* * Erythema* * nodosum* * is* * observed * *predominantly * *in * *women,* *whereas * *marked* * ankle* * periarticular * *inflammation * *or * *arthritis* * without* * erythema* * nodosum * *is * *more * *common in * *men [4]. This case fulfilled all the criteria of Löfgren's syndrome. The interesting feature in this patient is the reactivation of old scars.

Sarcoidosis affects the skin in 20%–30% of cases. Cutaneous sarcoidosis can manifest with specific and nonspecific lesions. The specific lesions are lupus pernio and plaques associated with more severe systemic involvement and more chronic course, while nonspecific lesions include erythema nodosum, the hallmark of acute and benign disease.

Scar sarcoidosis is a rare and highly specific manifestation of cutaneous sarcoidosis, with only a few case reports described. Scar sarcoid has not been reported in context of Löfgren syndrome.

Scar sarcoidosis presents with redness and recurrence of activity at the site of scars due to previous wounds, intramuscular injections, tattoos, herpes zoster, ritual scarification, or allergen extracts for desensitization. Itching is conspicuously absent which helps to distinguish it from clinical mimickers like hypertrophic scar and keloid. Scar sarcoidosis may herald a relapse or be a first presentation of acute sarcoidosis as in our case.

The severity of the systemic disease determines the need and modality of treatment. For patients with severe systemic involvement or disfiguring skin lesions, the mainstay of treatment is systemic corticosteroid therapy. Hydroxychloroquine and methotrexate are used as steroid sparing agents.

Recognition of cutaneous lesions is important because they provide a visible clue to diagnosis and serve to avoid more invasive attempts at obtaining tissue for histologic examination as in our case.
